# Parkin regulation of CHOP modulates susceptibility to cardiac endoplasmic reticulum stress

**DOI:** 10.1038/s41598-017-02339-2

**Published:** 2017-05-18

**Authors:** Kim Han, Shahin Hassanzadeh, Komudi Singh, Sara Menazza, Tiffany T. Nguyen, Mark V. Stevens, An Nguyen, Hong San, Stasia A. Anderson, Yongshun Lin, Jizhong Zou, Elizabeth Murphy, Michael N. Sack

**Affiliations:** 1 Cardiovascular and Pulmonary Branch, National Heart, Lung, and Blood Institute, National Institutes of Health, Bethesda, MD 20892 USA; 2 Systems Biology Center, National Heart, Lung, and Blood Institute, National Institutes of Health, Bethesda, MD 20892 USA; 3 Animal Surgery Program, National Heart, Lung, and Blood Institute, National Institutes of Health, Bethesda, MD 20892 USA; 4 MRI Imaging Core, National Heart, Lung, and Blood Institute, National Institutes of Health, Bethesda, MD 20892 USA; 5 Ipsc Core, National Heart, Lung, and Blood Institute, National Institutes of Health, Bethesda, MD 20892 USA

## Abstract

The regulatory control of cardiac endoplasmic reticulum (ER) stress is incompletely characterized. As ER stress signaling upregulates the E3-ubiquitin ligase Parkin, we investigated the role of Parkin in cardiac ER stress. Parkin knockout mice exposed to aortic constriction-induced cardiac pressure-overload or in response to systemic tunicamycin (TM) developed adverse ventricular remodeling with excessive levels of the ER regulatory C/EBP homologous protein CHOP. CHOP was identified as a Parkin substrate and its turnover was Parkin-dose and proteasome-dependent. Parkin depletion in cardiac HL-1 cells increased CHOP levels and enhanced susceptibility to TM-induced cell death. Parkin reconstitution rescued this phenotype and the contribution of excess CHOP to this ER stress injury was confirmed by reduction in TM-induced cell death when CHOP was depleted in Parkin knockdown cardiomyocytes. Isogenic Parkin mutant iPSC-derived cardiomyocytes showed exaggerated ER stress induced CHOP and apoptotic signatures and myocardium from subjects with dilated cardiomyopathy showed excessive Parkin and CHOP induction. This study identifies that Parkin functions to blunt excessive CHOP to prevent maladaptive ER stress-induced cell death and adverse cardiac ventricular remodeling. Additionally, Parkin is identified as a novel post-translational regulatory moderator of CHOP stability and uncovers an additional stress-modifying function of this E3-ubiquitin ligase.

## Introduction

The E3-ubiquitin ligase Parkin is enriched in multiple organs^1, 2^, resides in various subcellular locations^3, 4^ and its cognate ubiquitylation substrates modulate diverse cellular functions spanning from the regulation of gene transcription, protein stability, endoplasmic reticulum (ER) stress and mitochondrial quality control^5–8^. Given that mutations in the gene encoding for Parkin predispose to early onset Parkinson Disease^9^, our understanding of its regulatory functions in cellular homeostasis may shed insight into potential targets for the modulation of disease.

The most well characterized role of Parkin has been defined in the orchestration of adaptive mitochondrial autophagy (mitophagy) in the context of ‘de-energized’ mitochondria^10^. This adaptive Parkin-mediated mitophagy effect is evident in the heart following exposure to direct ischemia-reperfusion injury and following triggering of cardiac mitochondrial adaptive programing in response to ischemic preconditioning^11, 12^. Recently, Parkin has also been found to be essential in the turnover of mitochondria during cardiac development^13^. These studies highlight that: (i) the role of Parkin in protecting against mitochondrial stressors is operational *in-vivo*; (ii) Parkin plays an important role in mitochondrial maturation during cardiac development; and (iii) the heart may be a useful organ to explore additional putative regulatory roles of Parkin in response to biological stress.

We reasoned that cardiac pressure-overload biomechanical stress would be an additional pathology, to enable characterization of chronic Parkin-linked adaptive and/or maladaptive programming. One such chronically engaged program evident during cardiac hypertrophy is ER stress^14^. Interestingly, ER stress initially evokes adaptive programming, but when chronically engaged facilitate apoptosis^15, 16^. ER stress has been found to upregulate Parkin^17, 18^, and this E3-ligase is implicated in the modulation of ER stress protein folding^6, 19, 20^. To further characterize ER stress and to investigate the role of Parkin in this integrated stress response program we explored cardiac remodeling in response to thoracic aortic constriction (TAC) and in response to tunicamycin-activated ER stress in Parkin knockout (KO) mice.

We found that Parkin levels were robustly increased following TAC in wild type (WT) mice and that Parkin deficient mice exhibit exaggerated cardiac hypertrophy and contractile dysfunction in response to TAC. In parallel to this adverse remodeling, the C/EBP homologous protein (CHOP), a known regulator of ER stress initiated apoptosis, was disproportionally increased in Parkin KO mice. Furthermore, we identified CHOP as a Parkin ubiquitylation substrate and showed that CHOP turnover is regulated by Parkin. Additionally excess ER stress was evident in human primary fibroblasts from subjects with genetic mutations in *PARK2* and in Parkin deficient iPSC-derived cardiomyocytes. Together these data show that Parkin modulates ER stress via the post-translational control of CHOP levels to blunt adverse remodeling in response to pressure-overload and in response to systemic ER stress.

## Results

### Parkin depletion enhances cardiac hypertrophy and contractile dysfunction in response to pressure-overload

**Figure 1:**
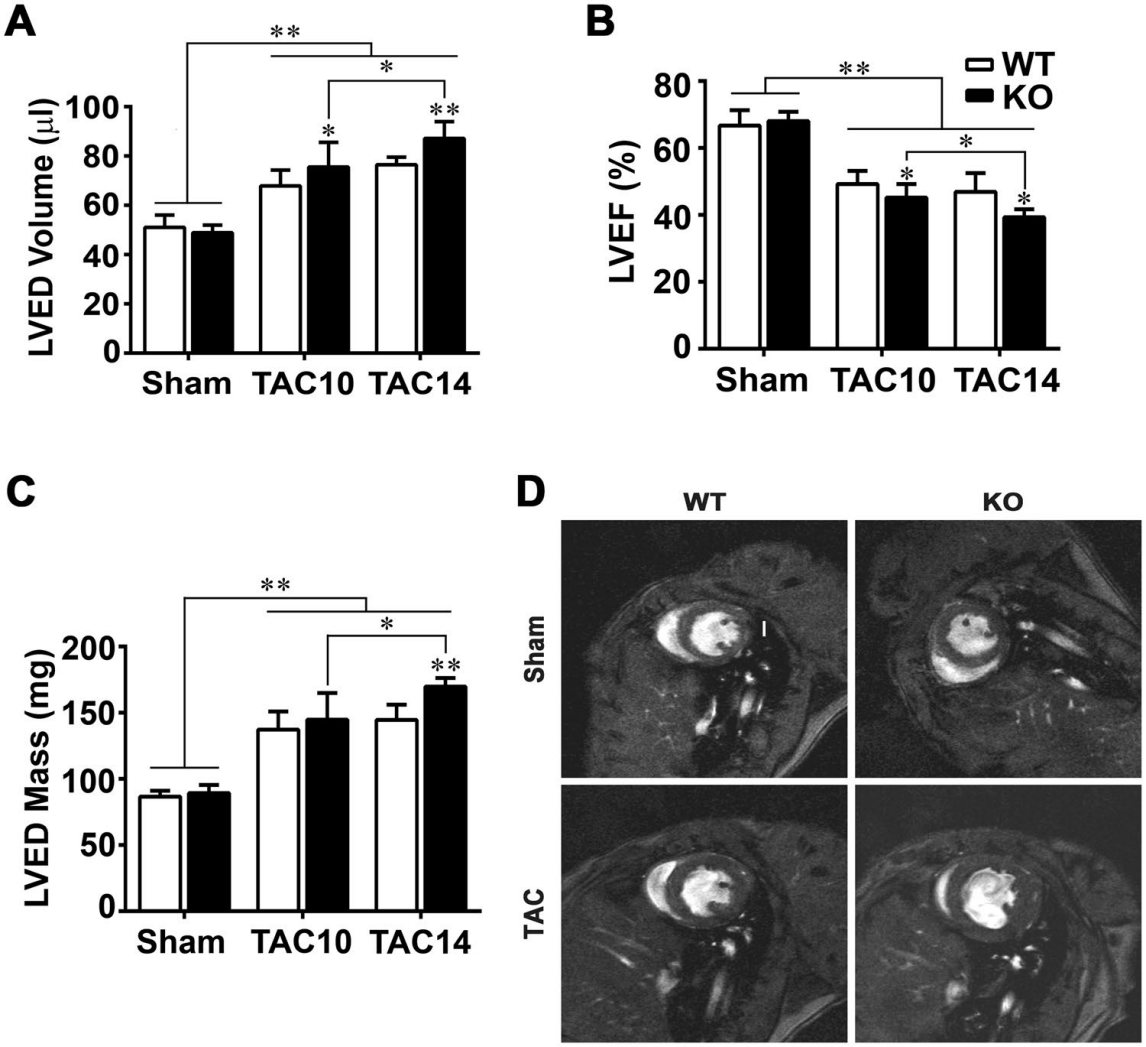
Adverse cardiac remodeling is exaggerated in the absence of Parkin. MRI quantification of (**A**) left ventricular end diastolic (LVED) volume, (**B**) LV ejection fraction (EF), and (**C**) LVED myocardial mass at 10 and 14 weeks following TAC. Data are shown as means ± SD (n = 6–12 per group). *p < 0.05 and **p < 0.01, compared to the corresponding controls. (**D**) Representative ventricular short-axis MRI images at end-diastole in mice at 14 weeks post-procedure. MRI Image was prepared 480 × 500 pixels resolution is 117 × 117 sq. micron in plane. The vertical white scale bar in the WT sham image represents 1-mm.

To explore the role of Parkin in adaptation to pressure-overload Parkin WT and KO mice were subjected to TAC followed by sequential imaging of cardiac structure and function over a 14-week period. By 10 weeks it was noted that the KO mice had evidence of exaggerated left ventricular dilatation (Fig. 1A) and worsening ejection fraction (45.14 ± 4.07% in KO-TAC vs. 49.24 ± 3.9% in WT-TAC) (Fig. 1B). By 14 weeks of TAC, the KO mice showed evidence of excessive hypertrophy and concomitant reduction in ejection fraction (39.32 ± 2.57% in KO-TAC vs. 46.91 ± 6.14% in WT-TAC) as measured by cardiac MRI (Fig. 1B–D). This measurement was confirmed by the direct assessment of cardiac mass indexed to tibial length (Supplemental Fig. S1A) and by histologic assessment of left-ventricular wall dimensions (Supplemental Fig. S1B). Quantifications of these changes are shown in supplemental Table S1. To assess whether the fetal gene program was differentially regulated in KO mice, transcript levels of genes encoding contractile proteins and cardiac natriuretic peptides were assessed. The fetal reprogramming of the contractile genes was activated to a similar extent in both strains with the development of cardiac hypertrophy (Supplemental Fig. S1C–F). However, in keeping with the increased ventricular dilatation, the transcripts encoding atrial natriuretic and brain natriuretic proteins (ANP and BNP), which are secreted in response to excessive chamber wall stress and fluid overload, were induced to a significantly greater extent in KO mice (Supplemental Fig. S1G,H).

### Diverse stress signatures are exaggerated following pressure-overload in Parkin KO mice

Oxidative stress and lipid peroxidation end products contribute to cardiac remodeling and play a role in ER stress signaling and apoptosis^21–23^. We therefore evaluated whether signatures of these modifications were modified in the absence of Parkin. We found that the extent of nitrotyrosine (Supplemental Fig. S2A,B) and 4-hydroxynonenal (Supplemental Fig. S2C,D) modifications of myocardial proteins were significantly higher in KO mice (2.6-fold and 2.0-fold vs. WT-TAC, respectively).

In light of the role of Parkin in stress-induced mitophagy, we explored mitophagy signatures in isolated mitochondria from sham and TAC mice. Here, the mitochondrial accumulation of an autophagy mediator (the lipidated form of the microtubule-associated protein 1A/1B-light chain 3 (LC3-II)) and the autophagy receptor (e.g. p62)^24^ were increased in mitochondria in response to TAC irrespective of the genotype. However, and consistent with the role of Parkin in mitophagy control the relative levels of accumulation of LC3-II and p62 were modestly reduced in the TAC KO mice (Supplemental Fig. S2E–G). Despite this, the mitochondrial content was not appreciably different, given that the mitochondrial DNA copy number (Supplemental Fig. S2H), and the levels of the mitochondrial proteins including medium chain acetyl-CoA dehydrogenase and cytochrome c were not differentially expressed in response to TAC comparing the two genotypes (Supplemental Fig. S2I,J). Additionally there was also no discernable phenotype difference in mitochondrial structural integrity in the sham or TAC mice by electron microscopy (Supplemental Fig. S2K).

### Pressure-overload induced ER stress is increased in Parkin KO mice hearts

**Figure 2:**
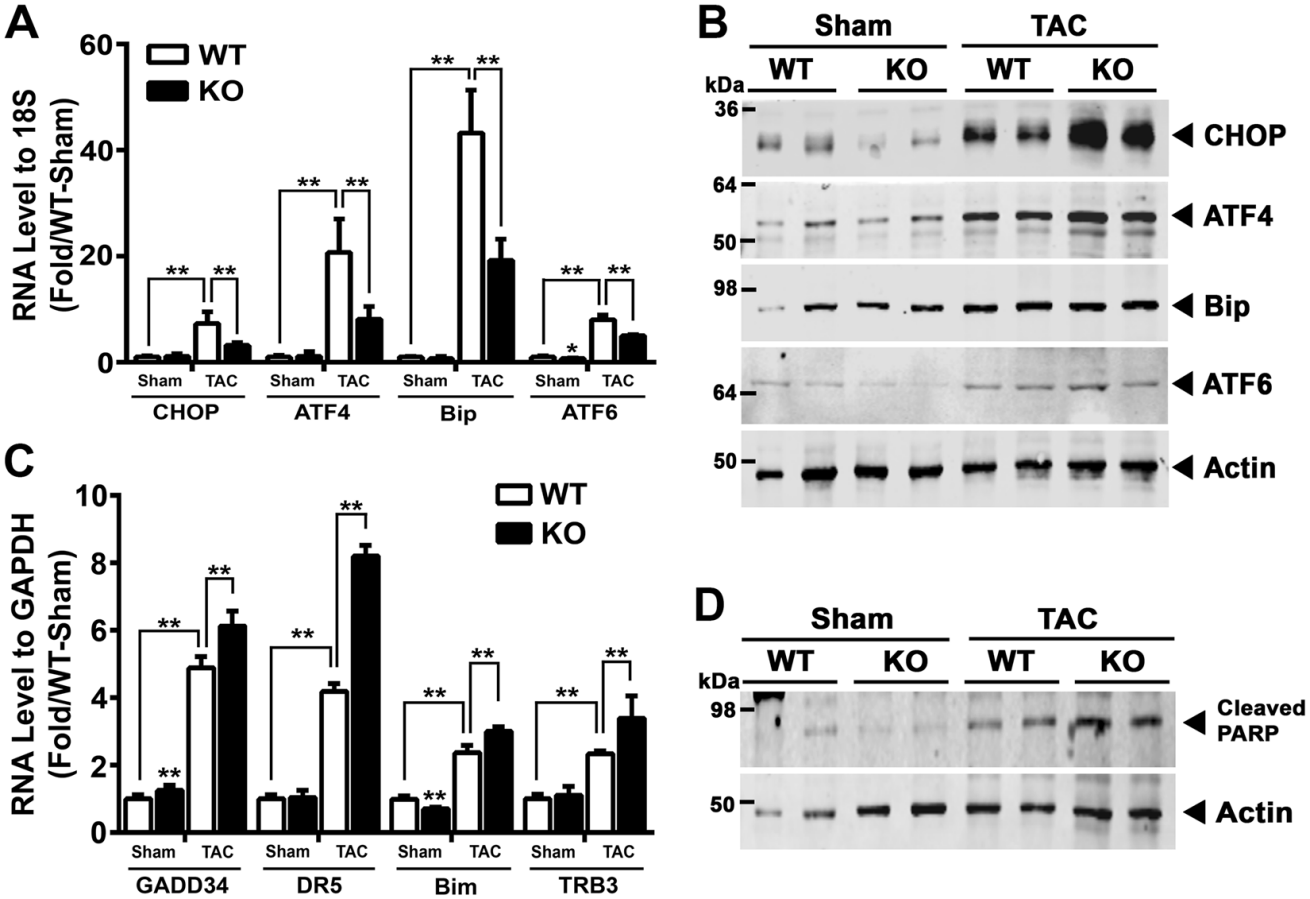
Parkin modulates ER stress during adverse remodeling in response to pressure-overload. (**A**) Quantification of ER stress responsive transcript (CHOP, ATF4, Bip and ATF6) levels in Parkin WT and KO mouse hearts in response to TAC. (**B**) Representative immunoblot showing expression of ER stress responsive proteins (CHOP, ATF4, Bip, and ATF6) in response to TAC. (**C**) RNA expression levels of CHOP target genes (GADD34, DR5, Bim and TRB3) following 14 weeks of TAC. Mean ± SD. **P < 0.01, vs. controls (n = 6 per group). (**D**) Representative immunoblot of cleaved PARP levels in Parkin WT and KO mouse hearts in response to TAC. The immunoblots in Fig. 2B and D are cropped images and the full membranes are depicted in a supplemental file.

To begin to explore ER stress directly we then assayed Parkin and ER stress levels in WT mice and found that Parkin and ER stress regulatory proteins were highly upregulated at the transcript and protein levels in response to TAC (Supplemental Fig. S3A,B). Comparing these data to the expression in Parkin KO hearts, it was interesting to note that the transcript levels of the genes encoding CHOP, Activating Transcription Factor 4 (ATF4), Bip/Grp78, and Activating Transcription Factor 6 (ATF6) were all significantly higher in WT compared to KO mice hearts in response to TAC (Fig. 2A). The steady-state protein levels of Bip/Grp78, ATF4 and cleaved ATF6 were similarly induced by pressure-overload in the two genotypes (Fig. 2B), as were the transcript level of spliced XBP1 (Supplemental Fig. 3C). In contrast, steady-state protein levels of ER stress regulatory protein CHOP was disproportionately more robustly increased in the TAC KO mice hearts (Fig. 2B and Supplemental Fig. S3D). As excess CHOP orchestrates maladaptive ER stress pathways^15^ and the genetic depletion of CHOP ameliorates maladaptive pressure-overload remodeling in the heart^25^, we evaluated whether cognate downstream transcriptional CHOP targets were differentially regulated in Parkin KO mice. Here we found the CHOP targets linked to apoptosis including GADD34, DR5, TRB3 and Bim^15^ were excessively induced at 14 weeks following TAC in the KO mice (Fig. 2C). Furthermore, as CHOP activation can result in adverse effects via PARP cleavage^26^, we assessed the extent of this modification in response to TAC. Consistent with the canonical upstream events, the extent of PARP cleavage was markedly higher in TAC Parkin KO mice (Fig. 2D and Supplemental Fig. S3E).

### CHOP is a Parkin substrate and its degradation is Parkin and ubiquitin proteasome dependent

**Figure 3:**
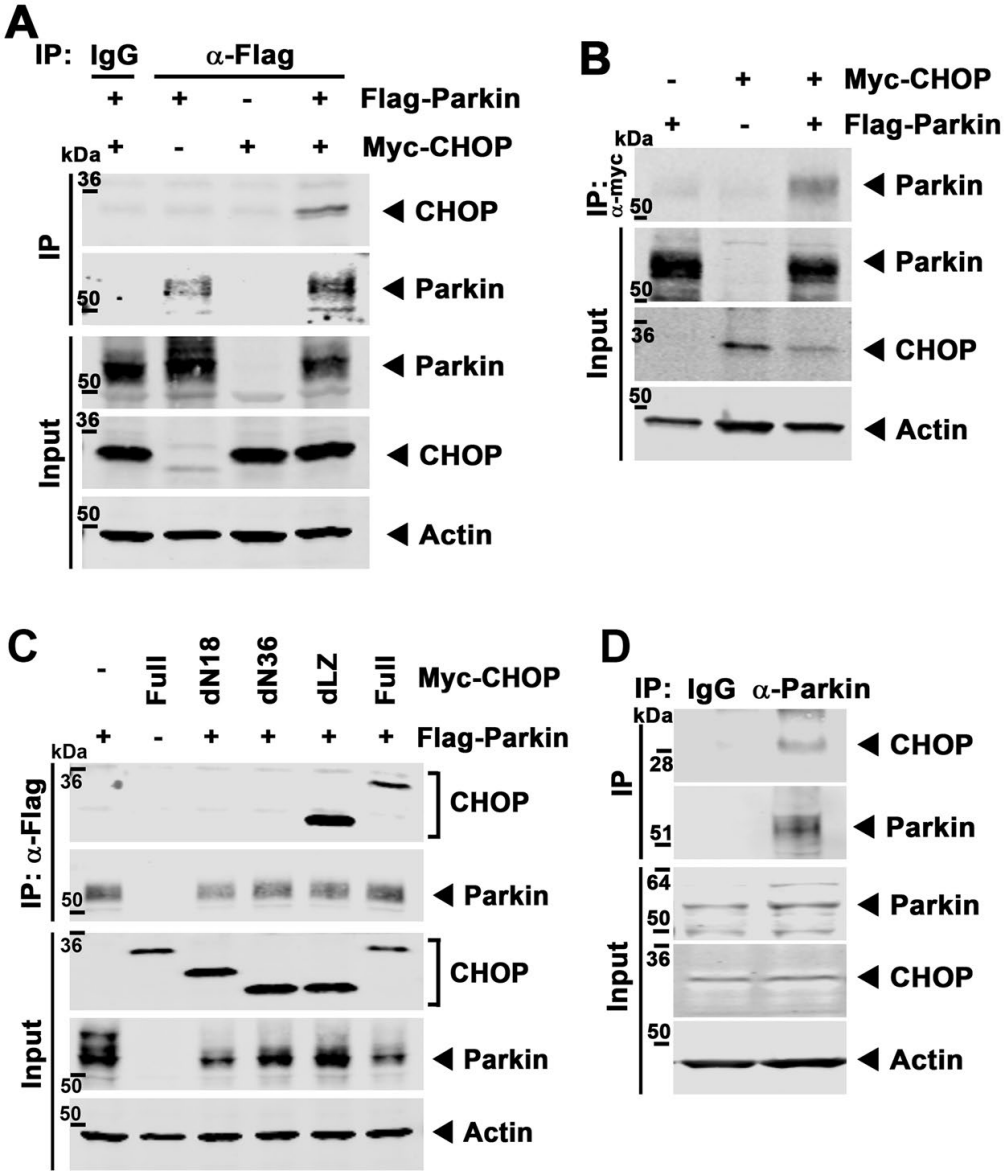
Parkin interacts with CHOP. (**A**) Representative gel by immunoprecipition with anti-Flag M2 agarose and immunoblotting with an anti-CHOP antibody in HL-1 cells transfected with pcDNA-Myc-CHOP constructs and p3 × Flag-Parkin. (**B**) Representative immunoblot with the reciprocal pull-down with anti-Myc agarose and immunoblot analysis with Parkin antibody. (**C**) Representative immunoblot for physical interaction in 293 cells transfected with the various deletion pcDNA-Myc-CHOP constructs and p3 × Flag-Parkin following immunoprecipitation with anti-Flag M2 agarose and immunoblot analysis with an anti-CHOP and Parkin antibody. dN18, 18-amino acid deletion of N-terminal CHOP; dN36, 36-amino acid deletion of N-terminal CHOP; dLZ, Leucine-Zipper domain deletion of CHOP. (**D**) Representative immunoblot with the endogenous protein interaction in thapsigargin-treated SH-SY5Y cells following pull-down with Parkin antibody and immunoblot analysis with CHOP and Parkin antibody. The immunoblots in this figure are cropped images and the full membranes are depicted in a supplemental file.

We next investigated the putative regulatory effects of Parkin on CHOP biology. We firstly assessed the protein interaction between Parkin and CHOP. Cotransfection and bidirectional co-immunoprecipitation studies showed robust interactions between CHOP and Parkin (Fig. 3A,B). Interestingly, the N-terminal alpha-helical domain of CHOP, which is known to bind to other regulatory proteins^27, 28^, was found to be the required domain for its interaction with Parkin (Fig. 3C). Morover, in high Parkin-expressing SH-SY5Y cells, the endogenous interaction of Parkin with CHOP was found in response to ER stress induction (Fig. 3D).

**Figure 4:**
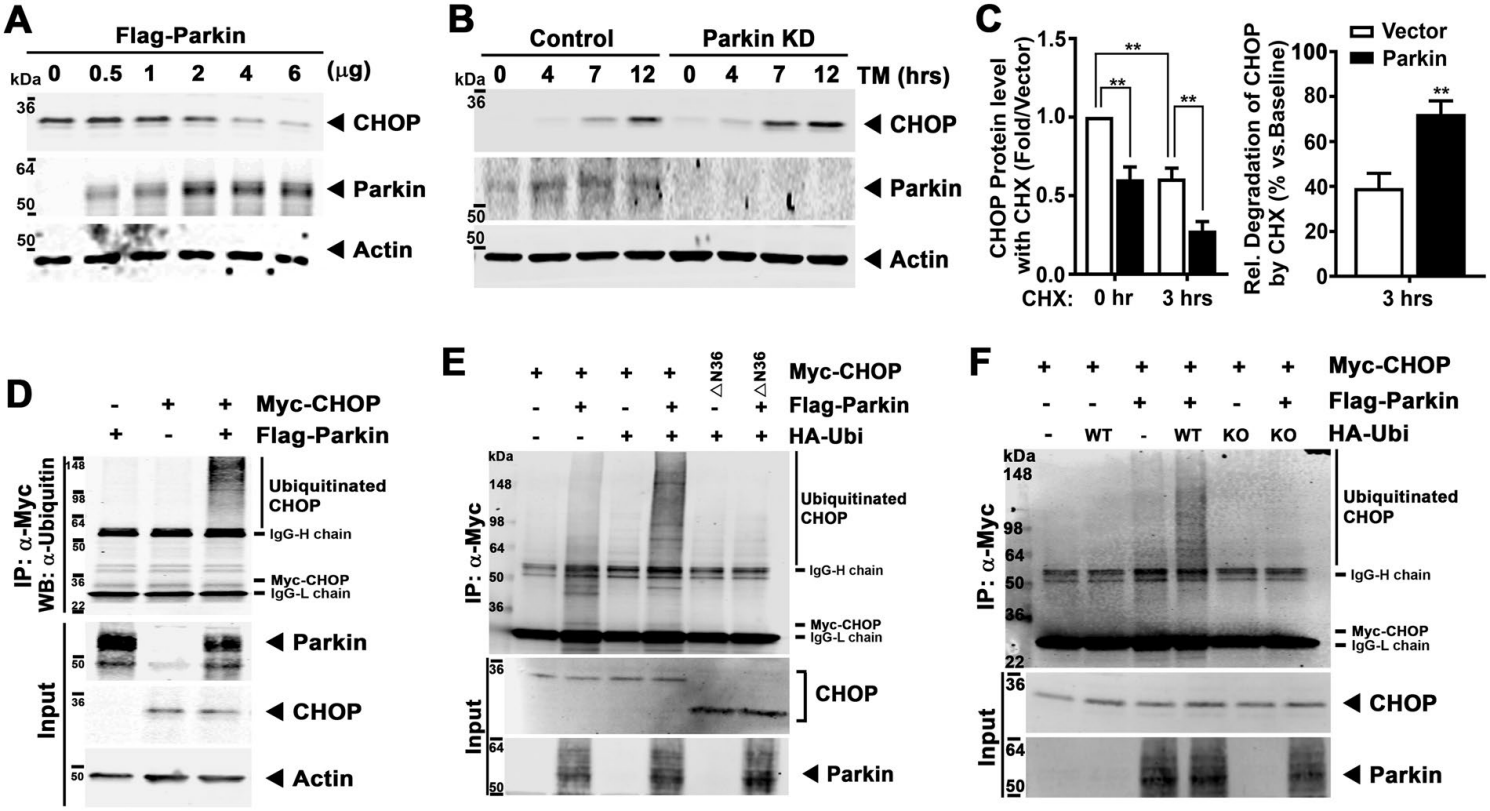
Parkin poly-ubiquitylates CHOP protein and modulates CHOP steady-state levels. (**A**) Degradation of endogenous CHOP with increasing doses of Parkin overexpression after TM administration for 12 hrs. (**B**) Immunoblot of temporal levels of endogenous CHOP in response to TM administration in control shRNA and Parkin shRNA HL-1 cells. (**C**) Quantification of endogenous CHOP protein level at 3 hrs of cycloheximide (CHX - 5 μg/ml) incubation in control and Parkin overexpressing cells compared to no CHX incubation. The cells are exposed to TM for 12 hrs followed by CHX. Mean ± SD. **P < 0.01, vs. controls. (**D**) Ubiquitylation of CHOP in HL-1 cells transfected with constructs of Myc-CHOP and Flag-Parkin following immunoprecipitation with anti-Myc agarose and immunoblot analysis with an ubiquitin antibody. (**E**) Ubiquitylation of CHOP in HL-1 cells transfected with constructs of Myc-CHOP, Flag-Parkin, and HA-Ubi (WT) following immunoprecipitation with anti-Myc agarose and immunoblot analysis with an ubiquitin antibody. △N36, 36-amino acid deletion of N-terminal CHOP. (**F**) Ubiquitylation of CHOP in HL-1 cells transfected with constructs of Myc-CHOP, Flag-Parkin, and HA-Ubi (WT and KO) following immunoprecipitation with anti-Myc agarose and immunoblot analysis with an ubiquitin antibody. HA-Ubi-KO, Lysine residues were substituted for arginine. The immunoblots in this Figure are cropped images and the full membranes are depicted in a supplemental file.

As TAC elicits excess CHOP in the absence of Parkin we then explored the effect of Parkin overexpression on endogenous CHOP levels in HL-1 cardiomyocytes. Here we found that Parkin, in a dose-dependent manner, progressively diminished steady-state levels of endogenous CHOP (Fig. 4A) without the modulation of the corresponding transcript levels (Supplemental Fig. S4A). To explore whether Parkin modulated CHOP protein levels in direct response to ER stress, Parkin shRNA HL-1 cells were exposed to TM. Here CHOP levels were more robustly induced in Parkin depleted cells (Fig. 4B and Supplemental Fig. S4B) without any difference in the induction of CHOP transcript levels (Supplemental Fig. S4C). To evaluate whether increased Parkin levels increased CHOP turnover, Parkin was overexpressed in TM treated cells in the presence or absence of the translational inhibitor, cycloheximide (CHX). In the presence of the CHX, the turnover of endogenous CHOP was significantly increased in the presence of Parkin (Fig. 4C and Supplemental Fig. S4D).

As Parkin is an E3-ubiquitin ligase we then explored whether the ubiquitylation of CHOP was modulated by Parkin. Immunoprecipitation of Myc-tagged CHOP with the subsequent immunoblot analysis with an ubiquitin antibody showed that excess Parkin increased ubiquitylation of CHOP with a pattern consistent of polyubiquitylation (Fig. 4D). Similarly, the co-expression of Parkin with ubiquitin, polyubiquitylated wildtype CHOP but not not the ΔN36 CHOP construct which we previously showed negated CHOP interaction with Parkin (Fig. 4E). In addition, the substitution of ubiquitin lysine residues with arginine similarly abolished Parkin mediated polyubiquitylation of CHOP (Fig. 4F). In parallel, and consistent with CHOP degradation by the ubiquitin-proteasome pathway, the exposure to the proteasome inhibitor MG132 increased CHOP levels in WT and Parkin KD cells. This being in contrast to the lack of increased CHOP levels by inhibition of the endosome/lysosomal degradation pathway by bafilomycin and/or chloroquine (Supplemental Fig. S4E,F).

### Parkin levels modulate cell death susceptibility in HL-1 cells

**Figure 5:**
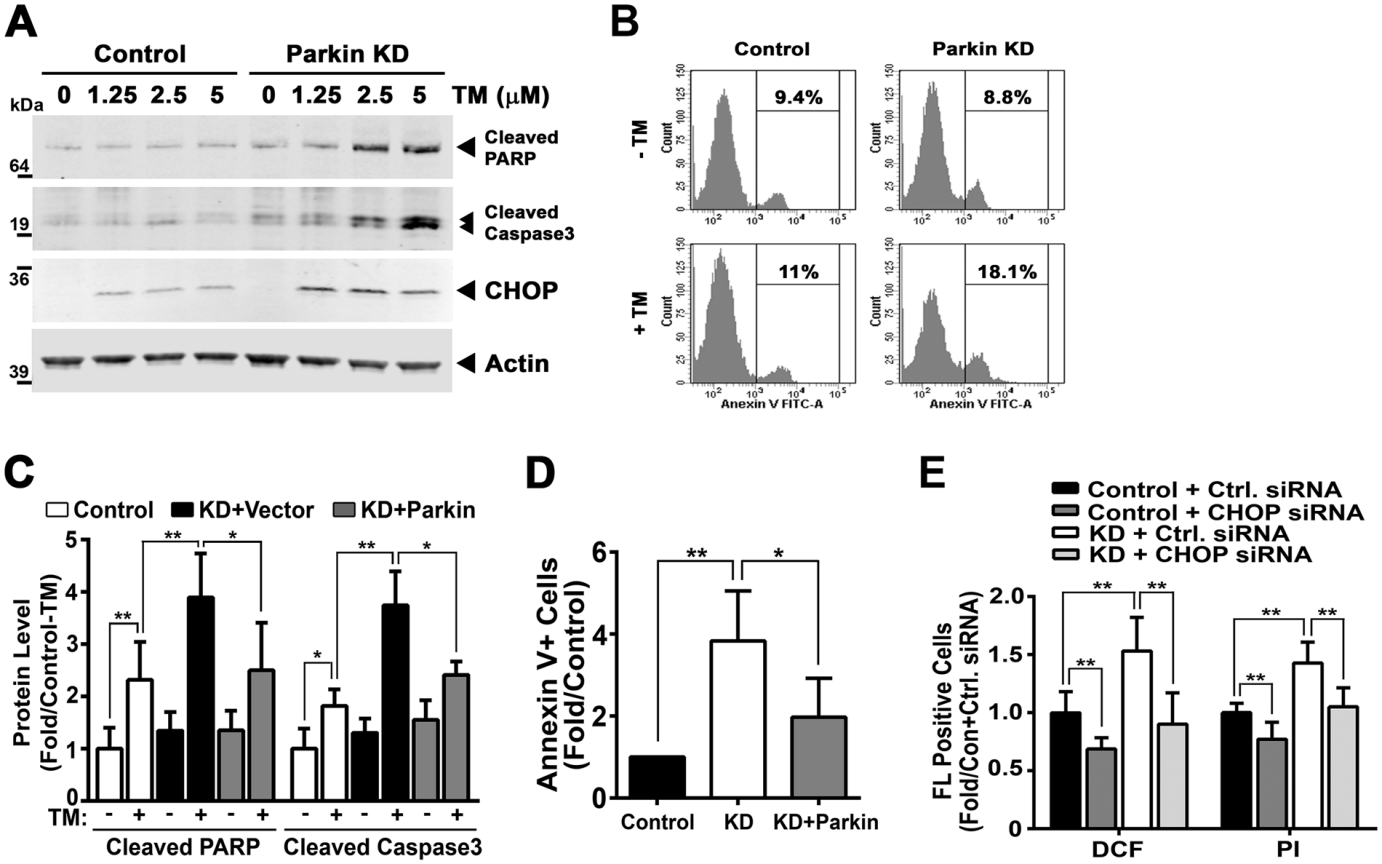
Parkin knockdown HL-1 cells to Tunicamycin show increased their susceptibility to apoptosis. (**A**) Immunoblot of the Cleaved PARP and Caspase3 protein after increasing doses of TM (0, 1.25, 2.5, and 5 μg/ml) for 24 hrs in control and Parkin KD HL-1 cells. (**B**) Representative flow cytometry image showing the increased Annexin V staining after TM administration (50 μg/ml) in Parkin KD cells vs. control shRNA HL-1 cells. (**C**) Quantification of PARP and caspase 3 cleavage level after reintroducing wildtype Parkin following the transfection of *2 µ*g of the Parkin expression plasmid. (**D**) Histogram showing the reduced Annexin V staining after TM in Parkin KD cells following the reconstitution of Parkin. (**E**) Histogram showing the relative reduction in DCF and PI staining in HL-1 cells in response to TM comparing Parkin to Parkin and CHOP knockdown. **P < 0.01, compared to the corresponding controls. The immunoblots in Fig. 5A are cropped images and the full membranes are depicted in a supplemental file.

Given the effect of Parkin on CHOP levels and following our observations of increased PARP cleavage in TAC Parkin KO hearts we then exposed HL-1 cardiomyocytes to TM to evaluate their susceptibility to apoptosis. Parkin knockdown HL-1 cells showed increased PARP and caspase 3 cleavage (Fig. 5A and Supplemental Fig. S5A) and increased annexin V staining vs. control shRNA HL-1 cells (Fig. 5B). The reconstitution of Parkin in the stable KD HL-1 cells diminished cell death as measured by the reduction in PARP and caspase 3 cleavage (Fig. 5C and Supplemental Fig. S5B,C), Annexin V staining, and reactive oxygen species levels compared to Parkin KD cells (Fig. 5D and Supplemental Fig. S5D,E). In parallel, the concurrent siRNA knockdown of CHOP in Parkin deplete HL-1 cells reversed the detrimental effects of tunicamycin on reactive oxygen species levels and cell death (Fig. 5E and Supplemental Fig. S5F,G).

### Cardiac dysfunction is exaggerated by ER stress in Parkin KO mice

**Figure 6:**
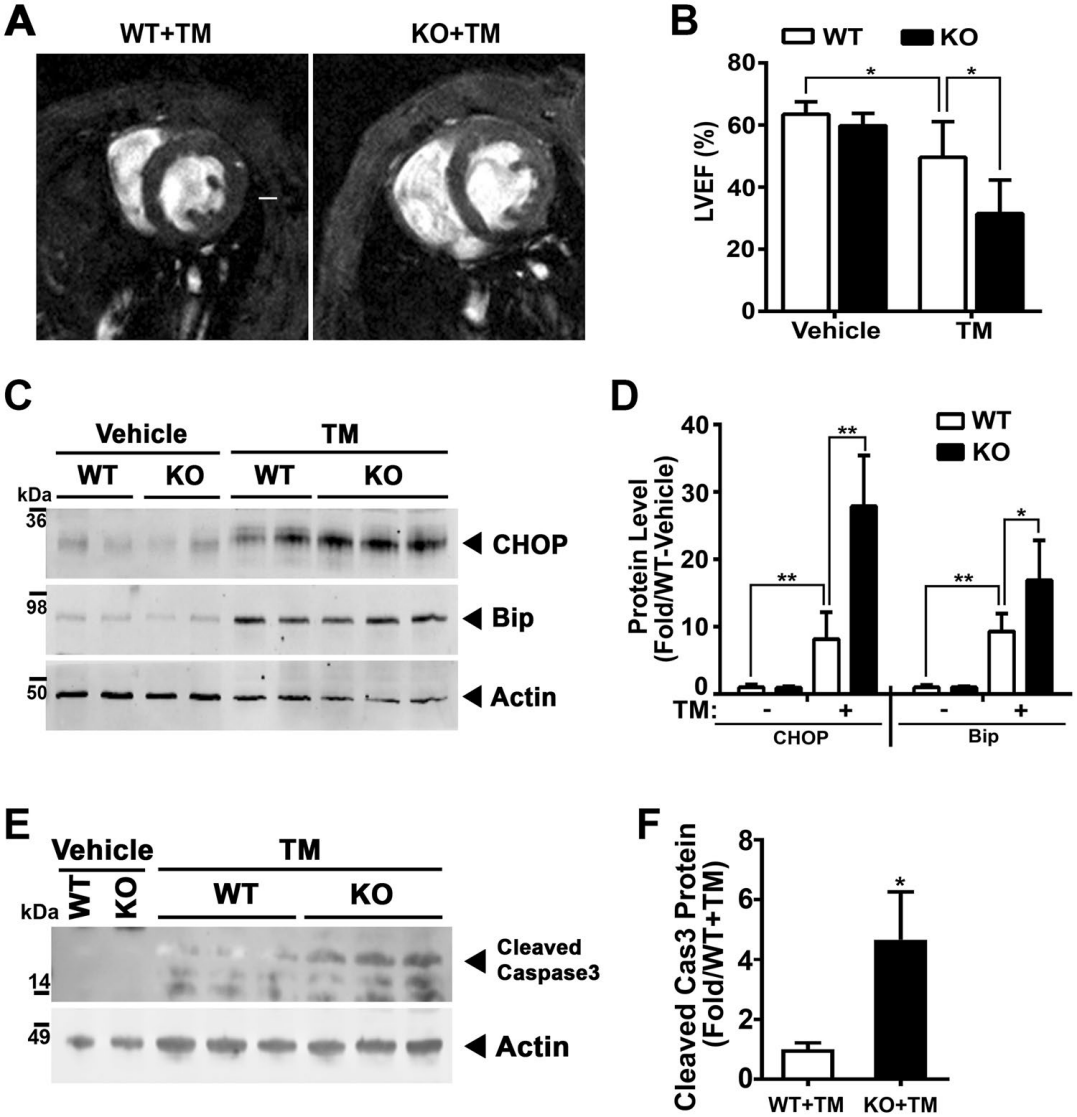
ER stress perturbations exacerbate cardiac dysfunction of Parkin KO mice. (**A**) Representative MRI image shows the cross section of heart of Parkin WT and KO mice 48 hrs. after systemic Tunicamycin (TM) administration. The white scale bar in the WT + TM image represents 1-mm. (**B**) MRI analysis of LVEF in WT and Parkin KO mice following the IP administration of Tunicamycin (2 mg/kg body weight). Mean ± SD. *P < 0.05, vs. controls. (n = 4–6 per group). Representative immunoblot (**C**) and histogram (**D**) showing the quantitation of CHOP and Bip/Grp78 in LV tissue extracted from the TM treated mice. (**E**) Representative immunoblot of the cleaved Caspase3 in LV tissue extracted from the TM treated mice. (**F**) Histogram showing the quantitation of cleaved Caspase 3. Mean ± SD. *p < 0.05 and **p < 0.01, compared to the WT-Vehicle controls (n = 4 per group). The immunoblots in Fig. 6C and E are cropped images and the full membranes are depicted in a supplemental file.

To directly evaluate whether ER stress itself differentially exacerbated cardiac dysfunction WT and Parkin KO mice were exposed to intraperitoneal TM. In concordance with the TAC studies, the KO mice showed greater left ventricular dilatation and a reduction in ejection fraction (Fig. 6A,B and Supplemental Table S2). This adverse remodeling was accompanied by higher levels of CHOP and Bip/Grp78 in the KO compared to WT murine hearts in response to TM administration (Fig. 6C,D). This effect also appeared to be due to the preferential induction of apoptosis, as measured by caspase 3 cleavage (Fig. 6E,F).

### Evidence of Parkin-dependent CHOP levels in human samples and the induction of this ER stress program in non-ischemic dilated cardiomyopathy

**Figure 7:**
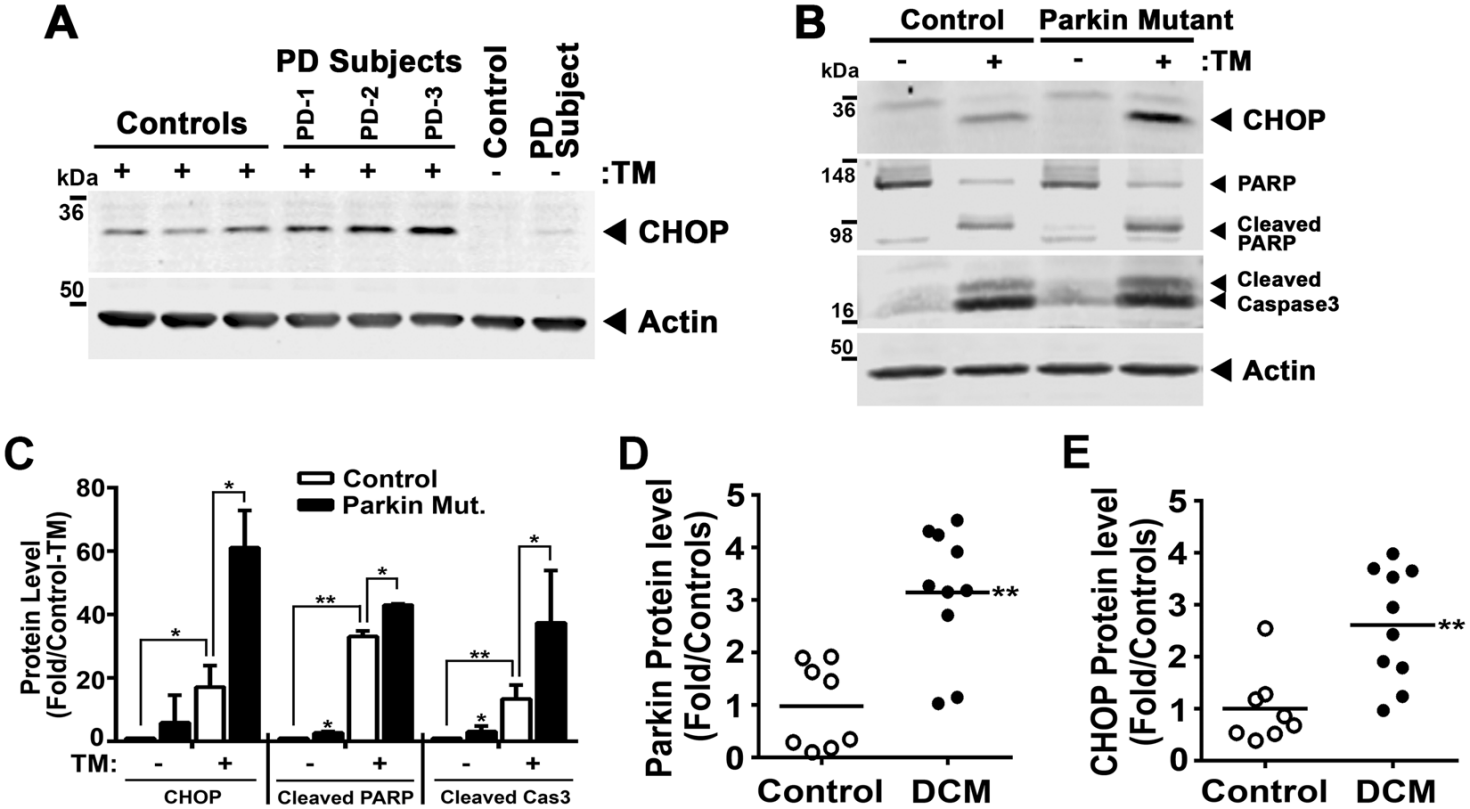
ER stress is evident in human Parkin mutant tissues. (**A**) Representative immunoblot of steady-state CHOP levels in controls and PARK2 mutant primary human fibroblasts exposed to TM (5 μg/ml) for 7 hrs. PD-1, deletion of Exon 3–4 and deletion of 255A; PD-2, mutation of GLN34ARG; PD-3, deletion of Exon 3 and mutation of ARG42PRO. Representative immunoblot (**B**) and histogram (**C**) showing the quantitation of CHOP Cleaved PARP, and Cleaved Caspase3 in the iPS cell-derived cardiomyocytes exposed to TM (5 μg/ml) for 48 hrs. Cardiomyocytes are differentiated from control and Parkin mutant induced pluripotent stem cells (iPSC’s). Data are shown as mean ± SD. *P < 0.05 and **P < 0.01, vs. control cells. Relative protein expression of Parkin (**D**) and CHOP (**E**) in 10 human dilated cardiomyopathy (DCM) heart tissues and 8 control subjects by immunoblot analysis. **P < 0.01, vs. control subjects. The immunoblots in Fig. 7A and B are cropped images and the full membranes are depicted in a supplemental file.

We then determined whether this role of Parkin is operational in skin fibroblasts extracted from individuals with early onset Parkinson disease with associated PARK2 mutations compared to control subjects. Basal CHOP levels were low and induced by TM with a significantly exaggerated CHOP induction in PARK2 mutant subject samples (Fig. 7A and Supplemental Fig. S6). Since Parkin levels are low in human fibroblasts, we then went on to assay whether this Parkin-CHOP regulatory interaction was operational in control and isogenic Parkin mutant inducible pluripotential stem cell (iPSC) derived cardiomyocytes. Here too, the disruption of Parkin resulted in elevated CHOP levels following exposure to TM in parallel with evidence of elevated PARP and Caspase-3 cleavage (Fig. 7B,C). The differentiation of iPSC’s into cardiomyocytes was evident by increased levels of the myocardial contractile protein including troponins I and T (Supplemental Fig. S7A). The CRISPR targeted disruption of Parkin was confirmed at both the sequence and steady-state protein levels (Supplemental Fig. S7B).

Finally, to evaluate whether this regulatory program may be operational in the human heart, we explored the protein levels of Parkin and CHOP in explanted hearts from unused control donor and from dilated cardiomyopathy patients. In parallel to the findings in WT mice in response to TAC, Parkin and CHOP levels were induced in the dilated cardiomyopathy heart samples, further supporting the activation of this program in human cardiomyopathy (Fig. 7D,E).

## Discussion

Cardiac remodeling in response to pressure-overload activates a wide array of efficiency and quality control programs including: alterations in contractile protein biology; the modulation of metabolic pathways to sustain bioenergetics and to control/ameliorate redox stressors; and the capacity for distinct organelles within the myocardium to orchestrate self-preservation and repair. Endoplasmic reticulum is one such organelle implicated in biomechanical stress adaptation, although mediators regulating the ER response are not as well characterized as other remodeling programs^29^. In concert with prior studies that implicate a role for Parkin in the control of ER biology, we showed that the absence of Parkin exacerbated adverse pressure-overload induced cardiac remodeling and evoked excess myocardial ER stress and apoptotic signatures. Additionally, Parkin was found to directly interact with, and modulate the protein stability of the pivotal ER stress regulatory protein CHOP. These findings identify a new Parkin substrate in the modulation of cardiac adaptation, and identify this E3-ubiquitin ligase in the post-translational regulatory control of ER stress.

The ER is responsible for protein folding, maturation, and trafficking, as well as functioning as a critical organelle in the regulation of cellular calcium homeostasis^30^. In response to cellular stressors, the ER can exhibit short-term adaptive effects, mediated in part by the ER stress response and/or, when stressful triggers are excessive or chronically engaged, perpetuate damage via ER stress signaling and apoptosis^15^. This biphasic role of ER stress is operational in the heart as evident by protection against acute ischemia-reperfusion injury following modest pharmacologic ER stress induction^31^ and where the genetic depletion of CHOP attenuates adverse remodeling following chronic pressure-overload^25^. The role of Parkin in ER stress biology has not been extensively characterized, although Parkin has previously been shown to be induced by ER stress^17^, and its upregulation is controlled, in part, by the ER stress activating transcription factor ATF4^18^. Additionally Parkin has been found to interact with CHIP and Hsp70 proteins to regulate ER protein folding^19^. In this study we found that the ER stress-induced transcription factor CHOP is a Parkin binding partner/substrate and that, in an ubiquitin/proteasome-dependent manner, Parkin modulates CHOP protein stability. Interestingly, adverse effects of CHOP can be alleviated when the adaptive pathways of ER stress are coordinately engaged^32^. In contrast to this parallel regulation, we found that in response to pressure-overload, the Parkin KO mice had diminished induction of the ER stress transcript levels with a disproportionate elevation in CHOP protein levels. In contrast, the induction of ATF4, ATF6 and Bip were similar between the WT and KO mice following TAC. These data, coupled with the *in-vitro* data showing that Parkin enhanced CHOP degradation and turnover in a dose-dependent manner, identify a new regulatory pathway in the control of chronic pressure-overload ER stress. The summation of these data support that the induction of Parkin in pressure-overload functions as an adaptive response to diminish CHOP levels to prevent/attenuate, in part, adverse ER-stress remodeling. Interestingly, additional regulatory proteins have been identified that control ubiquitin-proteosomal degradation of CHOP^28, 33^, suggesting potential context specific redundancy in the control of CHOP proteasomal degradation.

Parkin has previously been shown to mediate ameliorative effects via the attenuation of redox stress^34, 35^ and via the augmentation of mitochondrial autophagy^11, 12^. Although these adaptive programs were not the major focus of this study we did find evidence that these pathways were adversely affected in the absence of Parkin in response to chronic (14 weeks) cardiac pressure-overload. The apparent less robust contribution of mitophagy to this phenotype may reflect our temporal assessment of mitophagy signatures in this model. This is in contrast to where mitophagy had been found to be most active and important in the first week of cardiac adaptation to pressure overload^36^. At the same time it is conceivable that additional Parkin effects may be operational in cardiac remodeling in response to pressure-overload, including roles for Parkin in PARIS degradation as a mediator of mitochondrial biogenesis^8^, and/or in the maintenance of additional mitochondrial quality control programs^7, 37–39^. These pathways have not been investigated in this study and their role in this integrated adaptation would be useful to explore. In addition, and as opposed to the relatively extensive understanding of the regulatory control of Parkin activity in the mitochondria by the mitochondrial targeted PINK1^40, 41^, activation of Parkin’s extramitochondrial functioning has been less well characterized. Emerging data shows that the phosphorylation and nitrosylation of Parkin may modulate these extramitochondrial activity^42–44^. However, in this manuscript, we have not identified the regulatory control of Parkin activation in response to pressure-overload or ER stress.

**Figure 8:**
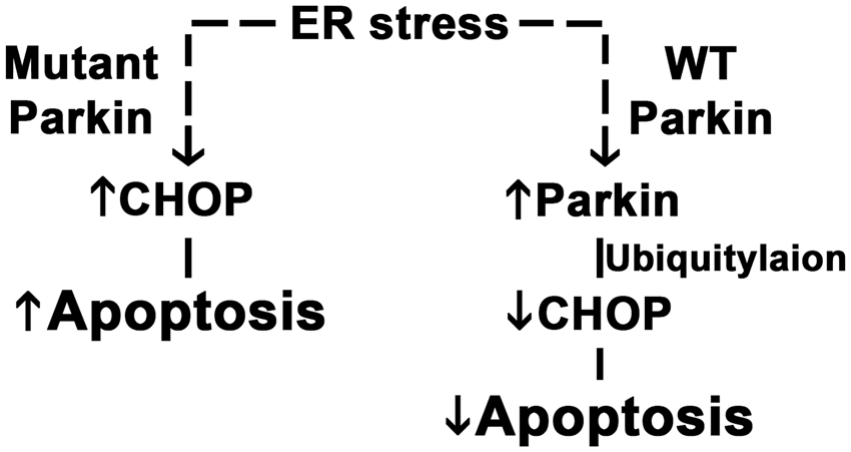
Working model of Parkin-mediated regulation during cardiac adaptation to pressure-overload.

The findings from this study expand our understanding of the regulatory role of ER stress in pressure-overload induced cardiac remodeling. We focused on the regulation of CHOP by Parkin, given that excessive CHOP is linked to maladaptive ER stress. Our data suggests that ER stress induction of Parkin in response to cardiac pressure-overload is an adaptive regulatory effect and that the role of Parkin in modulation of the steady-state levels of CHOP may function to maintain adaptive rather than progression to maladaptive ER stress signaling. This concept is further supported by the induction of Parkin and CHOP in cardiac samples from patients with dilated cardiomyopathy and evidence of the Parkin-dependent CHOP expression and evidence of increased apoptotic signatures in iPSC derived cardiomyocytes exposed to ER stress. However, the most compelling data supporting the role of Parkin in the blunting of ER stress is evident when WT and KO mice were directly exposed to systemic tunicamycin. Here, the reduction in ejection fraction and dilatation of the left ventricle was profoundly more evident in the absence of Parkin and these KO mice showed evidence of increased apoptosis. The model depicting the role of Parkin in this regulatory pathway is shown as a schematic (Fig. 8). At the same time we found that mediators of ER stress control of protein folding are also excessively induced in response to pressure overload (data not shown). Although Parkin had previously been shown to modulate protein folding^6, 19, 20^ it’s role of this ER-regulated program in cardiac hypertrophic pathophysiology remains to be explored.

We conclude that this study identifies a novel post-translational regulatory control node in the regulation of CHOP that, we postulate, may be operational to alleviate adverse effects of chronic ER stress activation. This program has been found to be active in response to cardiac pressure-overload and in human subjects with end-stage heart failure. Furthermore, these data identify an additional adaptive function of Parkin to attenuate adverse effects of chronic ER stress. Interestingly, ER stress is linked to Parkinson disease^19, 45^ and whether the loss of this ER stress regulatory control may contribute to increased susceptibility to early onset Parkinson disease in patients with mutations in *PARK2* is an intriguing concept that warrants investigation.

## Methods

### Animal Models and Human Tissues

Parkin knockout mice (129/Sv × C57BL/6 background) were obtained from the Jackson Laboratory and backcrossed (>10 generations) into the C57BL/6J strain. Tunicamycin (2 mg/kg body weight) was administrated by intraperitoneal injection and the cardiac contractile dysfunction was measured 48 hrs later. All animal procedures were conducted using an animal protocol that was approved by the National Heart Lung, and Blood Institute (NHLBI) Animal Care and Use Committee and all experiments were performed in accordance with NIH adopted National Research Council 2011 Guide for the Care and Use of Laboratory Animals. The diseased human heart samples were obtained from the Duke University Heart Transplant Program and the control samples from the National Disease Research Interchange (NDRI) tissue collection facility in Philadelphia, PA, USA. The National Institutes of Health (NIH) Office of Human Research Protections (OHRP) granted an exemption from IRB oversight for the scientific analysis of these deidentified human tissue samples.

### Transverse Aortic Constriction (TAC)

Parkin male mice at 14–15 weeks of age were subjected to TAC or sham surgeries. Briefly, the left chest of each mouse was opened to identify the thoracic aorta by blunt dissection at the second intercostal space after anesthetization with 1–3% Isoflurane and the absence of any toe pinch reflex. We then performed TAC using 7–0 silk sutures to band the thoracic aorta against 26-gauge needle for Parkin mice. The needle was removed before closing the thoracic cavity. A similar procedure, without constricting the aorta, was performed in the sham-operated group.

### Cell Culture Studies and Flow Cytometry

HL-1 cardiomyocytes (a gift from Dr. Claycomb) were cultured in 0.02% gelatin/12.5 mg/ml fibronectin-coated culture dishes with Claycomb medium (Sigma) containing 10% fetal bovine serum, 0.1 mM norepinephrine, and 2 mM L-glutamine with exchange of fresh media every three days^46^. 293 (human embryonic kidney cells) and SH-SY5Y (human neuroblastoma) cells were obtained from ATCC and the primary human fibroblasts from the Parkinson’s disease (PD) patients with *PARK2* mutation were purchased from Coriell Institute^47^. For flow cytometry analysis, HL-1 cells were incubated with Annexin V-FITC (Fluorescein isothiocyanate, eBioscience) and Propidium Iodide (PI, eBioscience) for 15 mins and 10 µM DCF (2′,7′-dichlorofluorescein, Invitrogen) for 1 hr, respectively and then were analyzed using the FACSCanto (BD).

### Isogenic iPSC-derived Cardiomyocytes

A gRNA sequence (TGCTAAGCGACAGG GGGTTC, followed by “CGG” PAM sequence) was selected from Exon 2 of human PARK2 gene by using online CRISPR design software at crispr.mit.edu with high ranking/specificity scores^48^. To enhance CRISPR/Cas9 editing efficiency, the gRNA sequence was cloned into a modified pX458 all-in-one CRISPR/Cas9 vector^49^, where CBh promoter was replaced with CAG promoter and the first nucleotide of gRNA was replaced with G^50^. Surveyor NHEJ assay was used to confirm that resultant PARK2-targeting CRISPR/Cas9 vector (pCAG458-PARK2-gRNA1) edited human 293T cells at the efficiency of ~39.5% (data not shown). Then 1 million ND1.4 human iPSCs were transfected with 5 ug pCAG458-PARK2-gRNA1 vector using lipofectamine 3000 (ThermoFisher) and following the manufacturer’s manual. Two days later, the GFP+ transfected iPSCs were sorted and plated on 10-cm Matrigel coated tissue culture plate at 10,000 cells/plate with 10 µM Rock inhibitor and E8 medium (ThermoFisher). Two weeks later, small single colonies were picked, expanded, and screened for PARK2 editing using PCR primers (F: 5′-gtttgcaggtcactgacgaa and R: 5′-ggaatccccaggcactattt). The gene-edited clones with homozygous or compound heterozygous indels were further confirmed by TOPO cloning and sequencing to select those with frame-shift indels that generate premature stop codon.

To induce cardiomyocyte differentiation from human iPSCs, 80% confluent iPSCs grown on Matrigel were cultured with basal differentiation medium (BDM) which contains E8 medium minus bFGF, TGF-β, and insulin, but plus 1X chemically defined lipid concentrate (Life Technologies) and 1x Pen Strep (Life Technologies). For the first 24 hrs of induction, 5 µM GSK3-beta inhibitor CHIR99021 (Tocris) was added to BDM. From day 2 to day 5, 3 µM Wnt inhibitor IWP2 (Tocris) and 3 µg/ml heparin (Sigma) were added to BDM. From day 7, >90% cells differentiated into beating cardiomyocytes expressing cardiac Troponin T and 20 µg/ml insulin (Sigma) was added to BDM to maintain cardiomyocytes with medium change every 2–3 days.

### Plasmids Transfection and Transduction

pcDNA plasmid encoding WT-Parkin was obtained as gifts from Dr. Olga Corti (Pitié-Salpêtrière Hospital, Paris, France) and then subcloned into p3xFlag vectors (Sigma). pcDNA-Myc-CHOP plasmids were purchased from Addgene and we then generated three deletion constructs of CHOP with 18-amino acid deletion of N-terminal (dN18), 36-amino acid deletion of N-terminal (dN36), and Leucine-Zipper domain deletion (dLZ). Transfection was performed using 1–2 µg of these plasmids with X-tremeGene HP (Roche) and Amaxa nucleofector (Lonza) according to manufacturer’s instructions. For stable knockdown of Parkin in HL-1 cells, cells were transducted with Lentivirus carrying mouse Parkin shRNA (SHCLNV-NM_016694, Sigma) and were selected with medium containing 1 µg/ml puromycin for 4 weeks. For knockdown of CHOP in HL-1 cells, cells were transfected with 400 pmol Control and Ddit3 SMARTpool siRNA (Dharmacon) with X-tremeGene siRNA (Roche).

### MRI Imaging

Cardiac MRI was used to quantify RV and LV function and morphology in the mice as previously described^51^. In brief, experiments were carried out in a 7.0T, 16 cm horizontal Bruker MR imaging system (Bruker) with Bruker ParaVision 5.1 software. Mice were anesthetized with 1.5–2.5% isoflurane and imaged with ECG and respiratory detection using a 35 mm m2m birdcage volume coil (m2m Imaging). Magnevist (Bayer Healthcare) diluted 1:10 with sterile 0.9% saline, was administered IV at a dose of 0.3 mmol Gd/kg body weight. Short axis gradient echo cine images of the heart were acquired (TR/TE = 10–11/3.4 ms, 4 averages, 1.0 mm slice thickness, 2.8–3.0 cm field of view, 256 × 256 matrix, 8–10 slices, respiratory and ECG-gated). Images were processed with CAAS MRV FARM software (Pie Medical Imaging) to determine left and right ventricle (LV and RV) ejection fraction, related functional parameters, LV and RV volumes and myocardial mass.

### Immunoblot and Immunoprecipitation analyses

Immunoprecipitation (IP) and immunoblot analysis were performed as described^2^. Total proteins from cells and tissues were extracted using RIPA buffer (50 mM Tris-HCl, pH 8.0, 0.5% deoxycholic acid, 1% NP-40, 0.1% sodium dodecyl sulfate and 0.5 M NaCl) supplemented with protease inhibitor cocktails and phosphatase inhibitors (Sigma). The lysates were separated by 4–20% Tris-Glycine or 4–12% Bis-Tris gel (Invitrogen) and transferred to nitrocellulose or PVDF membranes. Antibodies were purchased from Cell signaling (Parkin, Bip/Grp78, Ubiquitin, Nitrotyrosine, PARP, Caspase 3, VDAC, Cytochrome C, and GAPDH), Sigma (LC3), Progen (p62), Oxis international (4-hydroxynonenal, 4-HNE), Santa Cruz Biotechnology (CHOP, ATF4, Myc, and Troponin I), Novus (MCAD), Proteintech (ATF6), and Millipore (Actin). For immunoprecipitation (IP), cells were extracted using the lysis buffer (50 mM Tris-HCl, pH 7.4, 1% Triton X-100, 0.5% NP-40 and 300 mM NaCl) containing protease inhibitor cocktails and phosphatase inhibitors. Protein extract was incubated with antibodies against Myc or anti-Flag M2 agarose (Sigma)/anti-myc agarose (Pierce) overnight at 4 °C. For antibody conjugates, protein G agarose (Sigma) was added and incubated for 4 hrs. The agarose beads were washed with the lysis buffer and the supernatant with loading buffer was used for immunoblot analysis. For crosslinking protein interaction analysis, SH-SY5Y cells were treated with 25 mM thapsigargin for 5 hrs and crosslinked with 0.4% PFA for 20 mins on ice. The crosslink reaction was stopped with 125 mM glycine solution and the cells were lysed in Co-IP buffer (20 mM Tris-HCl pH 8, 137 mM NaCl, 1% NP-40, 2 mM EDTA, and 10% Glycerol). Parkin monoclonal antibody (Cell Signaling) and IgG antibody as control was added and incubated overnight at 4 °C. For antibody conjugates, sera-mag protein A/G magnetic beads (GE Healthcare Life Sciences) were incubated for 4 hrs and then washed with wash buffer (10 mM Tris-HCl pH 7.4, 1 mM EDTA, 1 mM EGTA, 150 mM NaCl, and 0.5% Triton X-100).

### Quantitative Real-time PCR

Quantitative real-time PCR was performed as described^47^. Total RNA was isolated from heart using Tripure isolation reagent (Roche). Reverse transcription of total RNA was performed using Superscrip III first-strand synthesis system (Invitrogen) according to the manufacturer’s protocol. Quantitative real-time PCR was performed using SYBR green PCR master mix (Applied Biosystems and Roche) and run on Opticon2 DNA engine (Bio-Rad) and Light cycler 96 systems (Roche). All reactions were normalized using GAPDH, TBP, or 18S. Primers were purchased from predesigned primers of QuantiTect primer assays (Qiagen)^52^.

### Statistical Analyses

Data are expressed as means ± SD for the indicated number of observations. Statistical significance between groups was determined using two-tailed Student’s test when analyzing the response between groups. Multiple comparison analysis was performed using ANOVA. *P* value < 0.05 was considered statistically significant.

## Electronic supplementary material

Supplemental Methods and Figures
