# Supplementary material for: Parkin regulation of CHOP modulates susceptibility to cardiac endoplasmic reticulum stress
Source: Sci Rep. 2017 May 18;7:2093. doi: 10.1038/s41598-017-02339-2 (PMC5437023; doi:10.1038/s41598-017-02339-2)
Supplement: Supplementary file 1 — Supplemental Methods and Figures [file 41598_2017_2339_MOESM1_ESM.pdf]

Parkin regulation of CHOP modulates susceptibility to cardiac endoplasmic reticulum stress

Kim Han[1], Shahin Hassanzadeh[1], Komudi Singh[1], Sara Menazza[2], Tiffany T. Nguyen[2], Mark V. Stevens[1], An Nguyen[1], Hong San[3], Stasia A. Anderson[4], Yongshun Lin[5], Jizhong Zou[5], Elizabeth Murphy[2], and Michael N. Sack[1,]

From the [1]Cardiovascular and Pulmonary Branch; [2]Systems Biology Center; [3]Animal Surgery Program; [4]MRI Imaging Core and the [5]iPSC Core (Y.L, J.Z.), National Heart, Lung, and Blood Institute, National Institutes of Health, Bethesda, MD, 20892, USA.

Online Methods

H&E Stain and TEM Imaging. After TAC for 14 weeks, mice were euthanized for isolating tissues. Left ventricle tissues were frozen in OCT compound, sectioned in 5 µm and stained with hematoxylin and eosin (H&E). Stained sections were examined by light microscopy (Olympus IX71). For electron micrographs, pieces of the left ventricle were fixed in 2.5% glutaraldehyde, 1% paraformaldehyde, 0.12 M sodium cacodylate buffer, pH 7.4 washed in cacodylate buffer, post-fixed in 1% OsO_4 in cacodylate buffer, stained en bloc with uranyl acetate, ethanol dehydrated, and Epon embedded. Thin sections were stained with uranyl acetate and lead citrate prior to imaging with a JEM1400 electron microscope (JEOL USA) equipped with an AMT XR-111 digital camera (Advanced Microscopy Techniques Corp).

Mitochondrial isolation. Heart mitochondria was isolated in isolation buffer (225 mM Manitol, 75 mM Sucrose, 1 mM EGTA, 5 mM HEPES, and 0.5% BSA, pH 7.4) using a Teflon/glass Dounce homogenizer and separated by differential centrifugation.

Mitochondrial DNA Copy Measurement. DNA was extracted from Parkin WT and KO mice with Sham and TAC using the Puregene Core Kit (Qiagen). Real-time PCR was performed for Cytochrome b (Cytb, Forward: 5′-CTTTGGGTCCCTTCTAGGAGTCTG-3′, Reverse: 5′-CGAAGAATCGGGTCAAGGTGGC-3′) and for the nuclear encoded 18S (Forward: 5′-CTTAGAGGGACAAGTGGCGTTC-3′, Reverse: 5′-CGCTGAGCCAGTCAGTGTAG-3′). Cytochrome b mitochondrial DNA copies were normalized to 18S.

Online Tables

Table S1. MRI analysis in WT and Parkin-deficient mice following Surgery

	Parkin WT			Parkin KO		
Parameters	Sham (n=6)	TAC10 (n=12)	TAC14 (n=6)	Sham (n=6)	TAC10 (n=8)	TAC14 (n=6)
LVEF (%)	66.72±4.61	49.24±3.90*	46.91±6.14†	69.04±2.80	45.14±4.07*†	39.32±2.57‡
LVED vol. (μl)	51.05±5.03	67.90±6.39*	76.45±3.39†	48.85±3.12	75.56±9.99*†	87.15±7.43‡
LVED mass (mg)	86.60±4.49	137.29±13.65*	144.56±12.68†	89.24±6.12	144.80±20.16*	169.77±7.10‡

TAC, Thoracic aortic constriction: TAC10, TAC for 10 weeks; TAC 14, TAC for 14 weeks; LVEF, Left ventricular ejection fraction; LVED vol., LV end-diastolic volume; LVED mass, LV end-diastolic mass. Results are presented as mean ± SD. *p<0.01 *vs.* Sham operation; †p<0.05 *vs.* WT TAC10; ‡p<0.05 *vs.* WT TAC14.

Table S2. MRI analysis in WT and Parkin-deficient mice following TM injection

	Parkin WT		Parkin KO	
Parameters	Vehicle (n=4)	TM (n=6)	Vehicle (n=4)	TM (n=6)
LVEF (%)	63.48±4.04	49.65±11.52*	59.83±4.02	31.53±10.83**†

TM, Tunicamycin injection was administered 48 hrs prior to the MRI; LVEF, Left ventricular ejection fraction. Results are presented as mean ± SD. *p<0.05 *vs.* WT-Vehicle; **p<0.05 *vs.* KO-Vehicle; †p<0.05 *vs.* WT-TM.

Online Figures

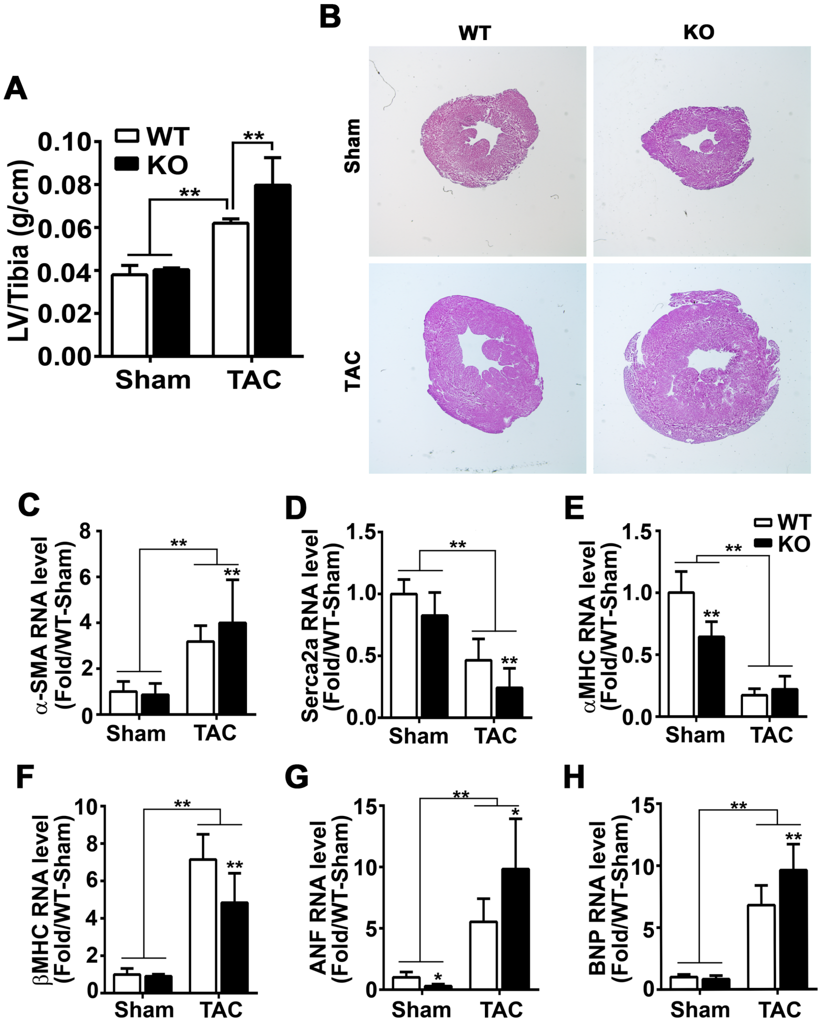

Figure S1. Contractile dysfunction is exacerbated in the absence of Parkin following TAC. (**A**) Post-mortem LV weight (g) and tibia length (cm) measured at 14 weeks after TAC. Data are shown as mean ± SD (n=6-12 per group). (**B**) Representative short-axis H&E sections of the mid-left ventricle at 14 weeks post-procedure. (**C-F**) Gene expression levels support in increased hemodynamic load in Parkin KO mice in response to TAC. RNA expression of hypertrophy-associated genes; α-SMA (**C**), Serca2a (**D**), α-MHC (**E**), and β-MHC (**F**) in LV tissue of Parkin WT and KO mice following 14 weeks of Sham or TAC surgery. (**G-H**) Transcripts of the ANF and BNP genes encoding cardiac secreted proteins in response to fluid/hemodynamic overload in Parkin WT and KO mice. Values represent the mRNA level relative to the GAPDH or 18S. Data are shown as means ± SD (n=6 per group). *P<0.05 and **P<0.01, compared to the corresponding controls. α-SMA, Alpha-smooth muscle actin; MHC, Myosin heavy chain; ANF, Atrial natriuretic factor; BNP, Brain natriuretic peptide.

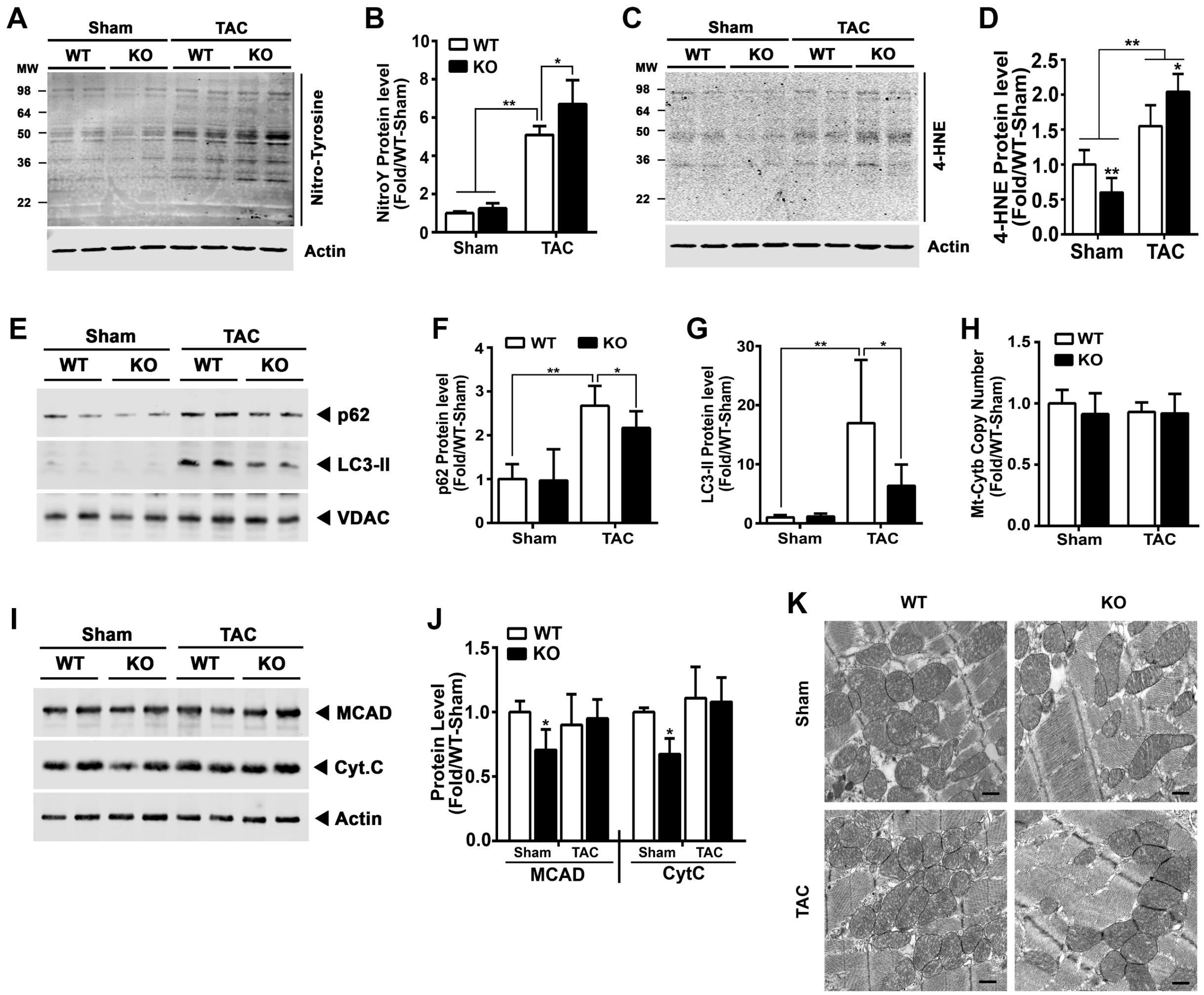

Figure S2. Parkin modulates redox and mitophagy during adverse remodeling. (**A-B**) Representative immunoblot showing nitrotyrosine levels indicative of oxidative stress with the corresponding quantification of the levels in response to 14 weeks of Sham and TAC in Parkin WT mice and KO mice. (**C-D**) Representative immunoblot showing 4-Hydroxynonenal (4-HNE) protein modifications with corresponding quantification of the levels. (**E-G**) Representative immunoblot and corresponding quantification of LC3-II and p62 expression in LV mitochondria of Parkin WT and KO mice following 10 weeks of surgery. (**H**) Histogram showing the mitochondrial cytochrome b DNA copy number (relative to 18s) in LV tissue of Parkin WT and KO mice following 14 weeks of surgery. (**I-J**) Representative immunoblot showing whole cell protein levels of MCAD and Cytochrome C with the corresponding quantification of the levels in response to Sham and TAC in Parkin WT mice and KO mice. (**K**) TEM micrographs of left ventricular cardiomyocytes of Parkin WT and KO mice following 10 weeks of surgery. Scale bar, 500 nm. All data are presented as mean ± SD. *p<0.05 and **p<0.01, compared to the corresponding controls (n=4-6 per group).

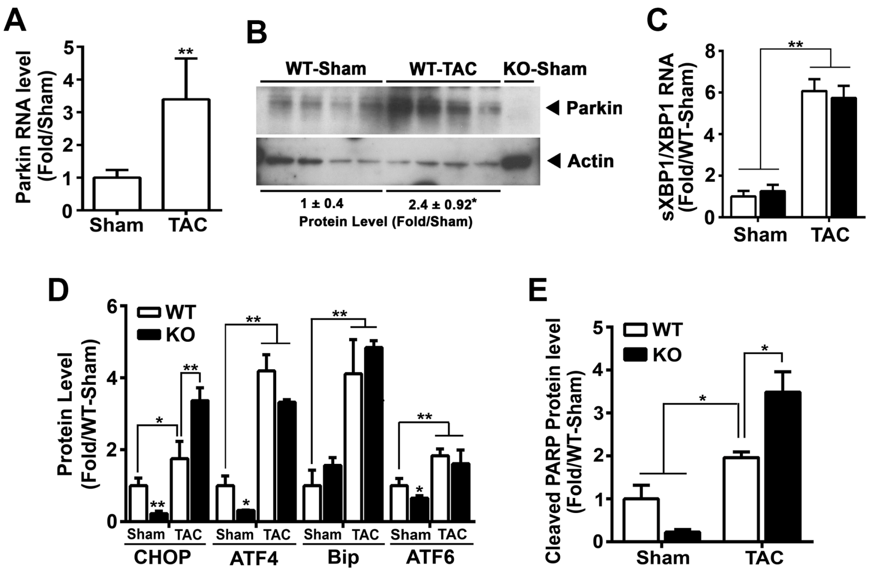

Figure S3. ER stress-related genes are induced by pressure-overload in WT and Parkin KO mice. (**A**) Parkin gene expression in response to 14 weeks of TAC in WT mice. Values represent the transcript levels relative to 18S. (**B**) Parkin protein expression and relative change in response to 14 weeks of TAC in WT mice. Mean ± SD. **P*<0.05, vs. sham controls (n=4 per group). (**C**) RNA expression of spliced XBP1 gene in LV tissue of Parkin WT and KO mice following 14 weeks of Sham or TAC surgery. Values represent the mRNA level relative to the XBP1. (**D**) Quantification of ER stress responsive protein (CHOP, ATF4, Bip and ATF6) levels in Parkin WT and KO mouse hearts in response to TAC. (**E**) Quantification of cleaved PARP levels in Parkin WT and KO mouse hearts in response to TAC. Data are shown as means ± SD (n=4-6 per group). *P<0.05 and **P<0.01, vs. the corresponding controls.

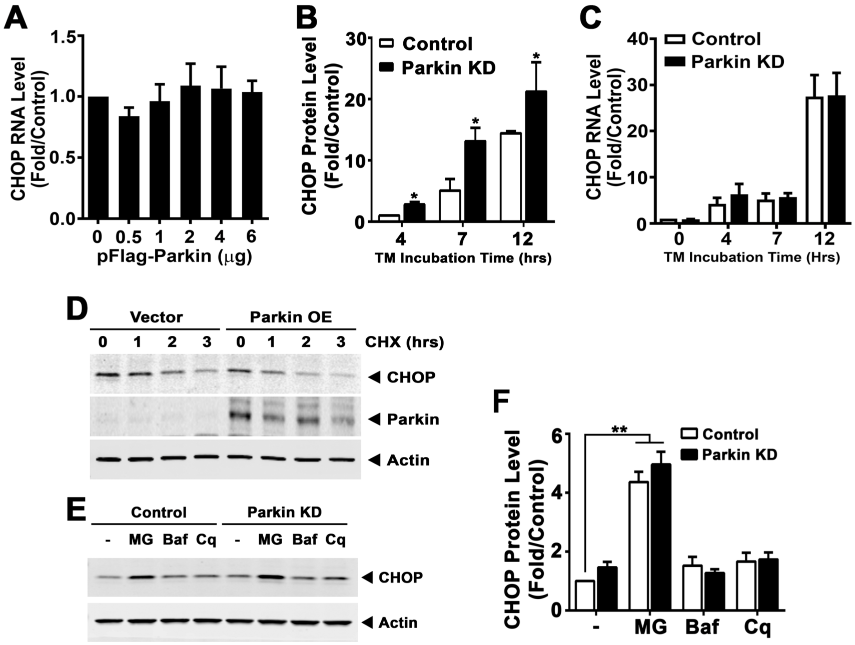

Figure S4. Parkin regulates the post-translational modification of CHOP. (A) CHOP gene expression with increasing doses of Parkin overexpression after TM administration for 12 hrs. Values represent the transcript levels relative to 18S. (**B-C**) Histogram quantifying the difference in CHOP protein and RNA levels after TM administration in control and Parkin KD cells, respectively. (**D**) Representative immunoblot showing the temporal degradation of CHOP protein levels in response to cycloheximide (CHX - 5 μg/ml) in control and Parkin overexpressing cells exposed to TM for 12 hrs followed by CHX. (**E**) The steady-state levels of CHOP were assessed in the presence or absence of Parkin following administration of MG132 (10 μM), Bafilomycin (50 nM), or Chloroquine (50 μM). (**F**) Quantification of CHOP protein levels in the presence or absence of Parkin with the administration of MG132 (MG), bafilomycin (Baf) or chloroquine (Cq), respectively. Data are shown as means ± SD. *p<0.05 and ***P*<0.01, compared to the corresponding controls.

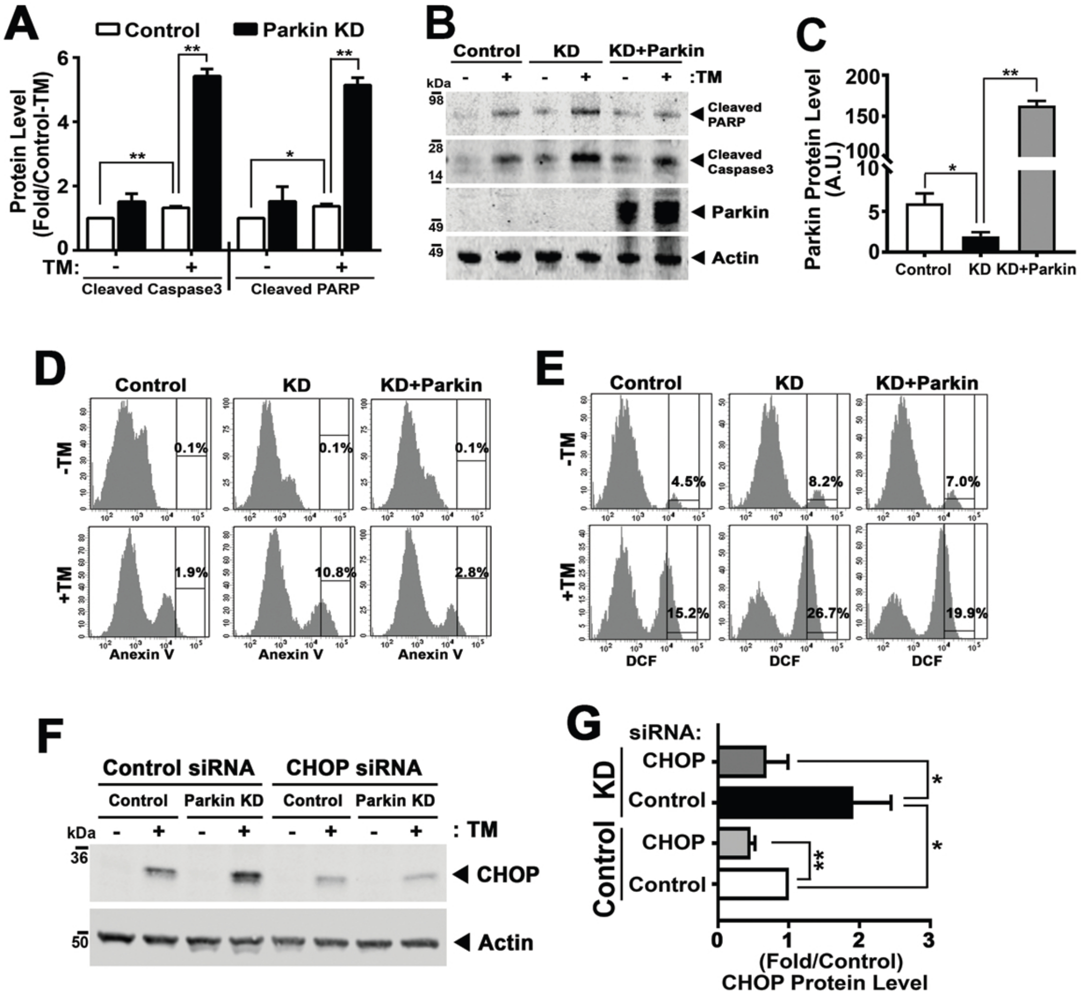

Figure S5. Parkin levels modulate apoptotic susceptibility in HL-1 cells. (**A**) Quantification of Cleaved Caspase3 and PARP protein levels after TM treatment (5 μg/ml) in control and Parkin KD cells. (**B)** Immunoblot of cleaved PARP, cleaved Caspase3 and Parkin protein levels after TM treatment in control and Parkin KD cells with and without Parkin reconstitution. (**C**) Quantification of Parkin protein level in HL-1 cells with and without Parkin reconstitution. Representative flow cytometry image showing Annexin V positive cell population (**D**) and DCF staining (**E**) after TM administration (50 μg/ml) in control and Parkin KD cells with and without Parkin reconstitution. Immunoblot (**F**) and histogram (**G**) showing the relative CHOP protein level in HL-1 cells in response to TM after CHOP knockdown, respectively. Data are shown as means ± SD. *p<0.05 and ***P*<0.01, compared to the corresponding controls.

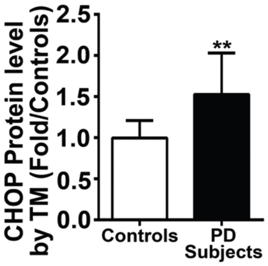

Figure S6. CHOP is increased in human PARK2 mutant cells. Histogram quantifying CHOP protein levels in control and Park2 mutant primary human fibroblasts exposed to TM (5 μg/ml) for 7 hrs. Values are normalized to GAPDH. Data are shown as mean ± SD (4 controls and 5 PD subjects). **P<0.01, vs. controls.

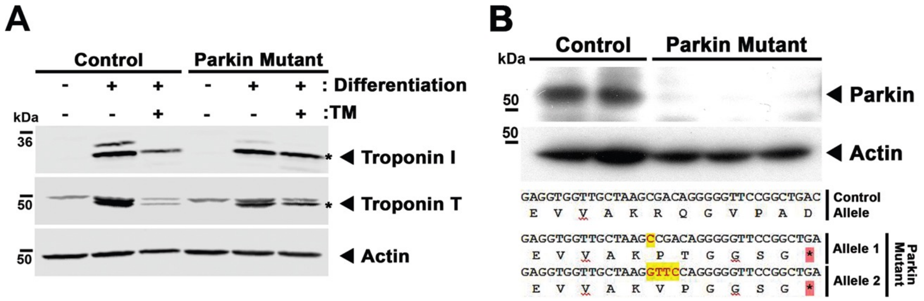

Figure S7. Evidence of cardiomyocyte differentiation and disruption of Parkin in inducible pluripotential stem cell (iPSC). (**A**) Immunoblot image showing Troponin I and Troponin T (as indicated with asterisks) expression in the iPSC's and iPS-derived cardiomyocytes with TM (5 μg/ml) for 48 hrs. (**B**) Representative immunoblot showing Parkin and validation sequencing data in the cardiomyocytes differentiated from control and Parkin mutant iPSC's. The nucleotide inserts in exon two on both alleles result in premature tga stop-codons as depicted by the asterisks in the downstream sequence.
